# Barriers and Facilitators to International Universal Health Coverage Reforms: A Realist Review

**DOI:** 10.34172/ijhpm.8709

**Published:** 2025-05-19

**Authors:** Liz Farsaci, Padraic Fleming, Louise Caffrey, Sara Van Belle, Catherine O’Donoghue, Arianna Almirall-Sanchez, David Mockler, Steve Thomas

**Affiliations:** ^1^Centre for Health Policy and Management, School of Medicine, Trinity College Dublin, Dublin, Ireland.; ^2^School of Social Work and Social Policy, Trinity College Dublin, Dublin, Ireland.; ^3^Institute of Tropical Medicine, Antwerp, Belgium.; ^4^Assistant Librarian Reader Services, Trinity College Dublin, Dublin, Ireland.

**Keywords:** Universal Health Coverage, UHC, Health Reform, Access, Implementation, Financial Protection

## Abstract

**Background:**

The journey towards universal health coverage (UHC) began decades ago but has recently moved to centre stage in global health discourses with its inclusion in the Sustainable Development Goals (SDGs). As part of this renewed interest, 193 countries have committed to introducing UHC by 2030. However, its implementation often necessitates far-reaching health system reforms. This, coupled with the struggles countries face in relation to health financing, as well as distinct political, social and cultural contexts, means there are significant challenges to UHC implementation. This article contributes new knowledge to these discourses by identifying key contexts and mechanisms that facilitate the successful implementation of UHC reforms, as well as barriers that can impede progress.

**Methods:**

This realist review identifies key contexts and mechanisms that can facilitate the successful implementation of UHC reforms. EMBASE, MEDLINE and Web of Science were searched (1995-2022), resulting in 957 articles with the protocol published through Prospero (PROSPERO 2023: CRD42023394427). Further theory-driven searches resulted in an additional 988 studies. Descriptive, inductive, deductive, and retroductive realist analysis aided the development of Context-Mechanism-Outcome Configurations (CMOCs), along with stakeholder engagement to confirm or refute results. Causal pathways, and the interplay between contexts and mechanisms that triggered outcomes, were revealed.

**Results:**

How each country goes about implementing UHC reforms depends on its context. Cohesion across all systems, as well as the functions of financing, governance and service delivery, facilitates these reforms. Implementation can also be facilitated through political commitment, communication between stakeholders in the public health system and the development of a strong primary care sector. Conversely, fragmentation across these functions pose significant barriers to UHC reforms.

**Conclusion:**

Examining international experiences of UHC reforms supports learning around the mechanisms that support or hinder implementation processes. These learnings can empower policy-makers and health system leaders by providing roadmaps for reform implementation.

## Background

 Health systems around the world are moving towards universal health coverage (UHC), which is included within the Sustainable Development Goals (SDGs), and which 193 countries have committed to introducing by 2030. UHC has been promoted as a solution that can strengthen health systems, raise revenue for healthcare and improve risk protection.^1^ The World Health Organization (WHO) has been a driving force behind these reforms, and many countries use the WHO Cube for UHC as a framework for developing the breadth, depth and scope of services provided.^2-4^

 While there has been renewed international interest in UHC in recent years, countries have introduced UHC at different times throughout the past century. Following World War II, a number of European countries made progress towards UHC in the form of national health services within the context of a desire for social cohesion. The move towards universalism was mirrored in Canada, which introduced UHC in the late 1940s as part of broader social reforms. More recently, countries in Latin America introduced health reforms as part of larger political and social movements. In 1978, 134 member states of the WHO ratified the Declaration of Alma-Ata, committing to improving health through strengthening primary healthcare (PHC), a crucial component of UHC. Since the millennium, health system reforms have also occurred across a wide range of both low- and middle-income countries (LMICs)^5-8^ and high-income countries (HICs).^9-11^

 However, because the implementation of UHC often includes far-reaching, system-wide change—as well as the redistribution of resources and power^1^—countries can face a number of challenges along the path to reform. How individual countries go about establishing UHC or scaling up their national health systems toward universal coverage depends on the context of each country and how their health systems are organised and financed. Furthermore, these far-reaching reforms often include a significant reorganisation of a system’s governance structure.^12^

 This paper adds new knowledge to discussions regarding the implementation of UHC reforms through highlighting contexts and mechanisms that can facilitate the successful implementation of these reforms, as well as the barriers that can impede them. Through examining the kaleidoscope of contexts and mechanisms that have facilitated successful UHC reform implementation in countries throughout the world, our research offers both a direction of travel and a roadmap for policy-makers and public health system leaders charged with bringing greater universality to their populations.

 It is important to note that there is an absence of uniformity around the conceptual definition of UHC.^13^ However, these dialogues remain largely outside the scope of this study. For the purpose of this review, we have adopted the definition of UHC as defined by the SDG 3.8 target: “Achieving UHC, including financial risk protection, access to quality, essential healthcare services and access to safe, effective, quality and affordable essential medicines and vaccines for all.”^14^

## Aims and Objectives

 The aim of this realist review was to identify key contexts and mechanisms internationally that can facilitate the successful introduction and implementation of UHC reforms or act as barriers within national public health systems.

## Methods

 The aim of a realist review is to generate causal explanations for observed outcomes within the context of a complex phenomenon. The realist approach allows researchers to develop explanations for how and why things happen and has become a popular tool for investigating complex health system issues.^15^ As health systems around the world work to introduce or implement UHC, they are contending with diverse contexts and outcomes. Therefore, a realist approach was deemed appropriate as part of an attempt to understand UHC policy development and implementation.

**Figure 1:**
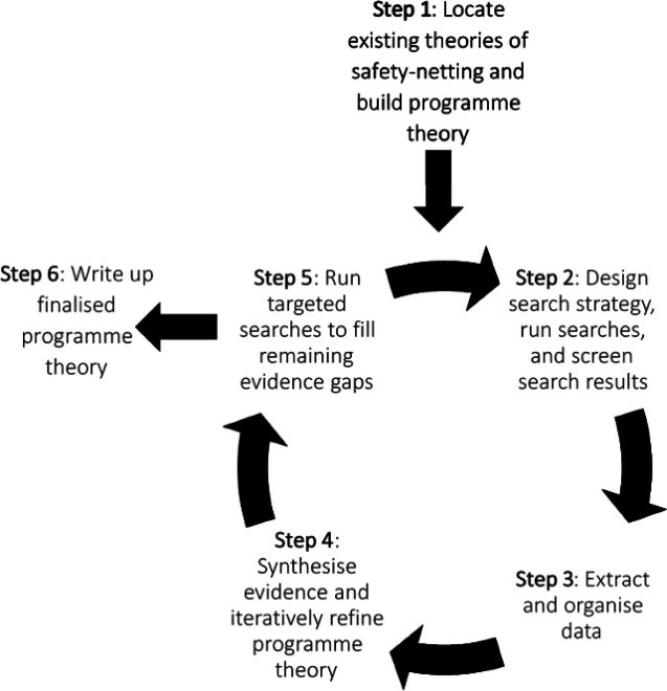
The Six Steps of a Realist Review.^18^

 Specifically, realist reviews deepen understanding of the causal explanations for observed outcomes through the development of a programme theory. They are inherently mixed methods studies,^16,17^ with qualitative, quantitative and mixed methods studies, as well as grey literature, all considered to be valuable sources of data if they can assist in theory building.^15^ This theory is produced through the development of context-mechanism-outcome configurations (CMOCs), which are informed by the data included within the review. The process which a realist review follows is illustrated in Figure 1.^18^

 In order to actualise this process and establish trustworthiness and rigour, this 6-stage approach was taken^16^:

 1. Initial Programme Theory (IPT) development

 2. Formal literature searching based on IPT

 3. Data screening and extraction

 4. Data analysis and preliminary CMOC development

 5. Additional literature searches to refine CMOCs

 6. Development of refined CMOCs and a finalised Programme Theory

###  Stage 1: Initial Programme Theory Development

 An IPT was developed based on informal reading and discussions that emerged from a stakeholder focus group held in December 2022. The focus group, which included six experts in total, was composed of national and international health system and policy analysts, one of whom is also an expert in realist methodology, a senior policy-maker from the Ministry of Health and a Public and Patient Involvement representative. The final member of the group is a primary care expert and healthcare worker. These stakeholders provided feedback to the research team regarding factors that most significantly impact the success of health reforms and influence implementation and policy design changes. This advice informed the key search terms utilised within the formal literature search.^19^

###  Stage 2: Formal Literature Searching Based on Initial Programme Theory 

 Following the informal or scoping search of the literature outlined above, the “main” search of the evidence included a systematic search of academic databases using key search terms. With the assistance of a library specialist (DM), an initial search strategy was developed and conducted in line with published protocol.^19^ The research spanned from 1995 to 2022 across three databases (EMBASE, MEDLINE, Web of Science). The original inclusion and exclusion criteria were based on relevance to the IPT. Per these criteria, the search focused on reforms taking place within the public health system, as this review is situated within the larger RESTORE project, which is investigating reform and resilience within public health systems. The role of the private sector in reforms was outside the scope of this review.

###  Changes Since Published Protocol

 The inclusion and exclusion criteria were amended following the publication of the protocol.^19^ Per the protocol, articles discussing national public health system reforms were included. However, following the initial search and abstract screening, the inclusion criteria was amended to only include articles addressing UHC reforms specifically, given the interest of the larger RESTORE project and the global health community on UHC reforms, and for reasons of feasibility. Outside this amendment, the inclusion and exclusion criteria remained the same. The amended inclusion and exclusion criteria are provided in Table.

**Table:** Eligibility Criteria

**Inclusion Criteria **	**Exclusion Criteria **
The studies must focus on national UHC reforms	Studies not focusing on UHC reforms
Studies that focus on disease specific outcomes, for example, must be in the context of system-wide UHC reforms	Studies focusing on regional or geographically siloed reform initiatives
The reform policy must have been approved and adopted by government	Studies focusing on programme specific reforms, independent of wider health system reforms
The study can include pre-implementation research once the policy has been officially adopted by government	Studies that focus on individual pillars of health system reform, eg, governance or finance, that do not have implications for broader health system reform
The study must relate to reform integrity, implementation or evolution	Studies focusing on private healthcare only with no consideration for knock-on impact for public system

Abbreviation: UHC, universal health coverage.

###  Stage 3: Data Screening and Extraction

 EMBASE, MEDLINE, and Web of Science were searched, resulting in 957 articles. After abstract screening, a total of 376 articles were included, while 163 articles were included after a full text screening. At this point in the project, the decision was taken by the research team to only include articles addressing UHC reforms, as outlined above. A full text screening based on this additional inclusion/exclusion criteria yielded 27 articles, although the final number was 24, as three articles were later excluded due to lack of relevance. See Supplementary file 1 for included articles.

###  Stage 4: Data Analysis and Preliminary CMOC Development

 Data from included studies were extracted using NVivo qualitative analysis software. Deductive analysis was conducted in the first instance using a coding tree based on the IPT, followed by inductive (data driven) analysis, which enabled the data to be organised into five themes. Retroductive analysis, which can offer causal explanations beyond the logics of deductive and inductive analysis,^20,21^ was then conducted. Retroductive analysis—“the activity of unearthing causal mechanisms”^21^ (p. 121)—allows the researcher to identify mechanisms that lead to observed patterns within the data, with a view to developing middle range theories in the form of CMOCs. In order to operationalise this, the lead author familiarised herself with the data through leading the inductive and deductive analysis. She then re-read the data extracted from this initial analysis and suggested to the co-authors potential mechanisms that were triggered within certain conducive contexts to produce the outcomes discussed within the data. ST also examined a portion of the data, in order to test these potential mechanisms and help distinguish between latent, or universal, mechanisms and activated, or context-specific, mechanisms.^21^ This process yielded 50 initial CMOCs, which the authors then tested through an iterative process of discussion and re-writing, consolidating them into 11 CMOCs.

###  Stage 5: Additional Literature Searches

**Figure 2:**
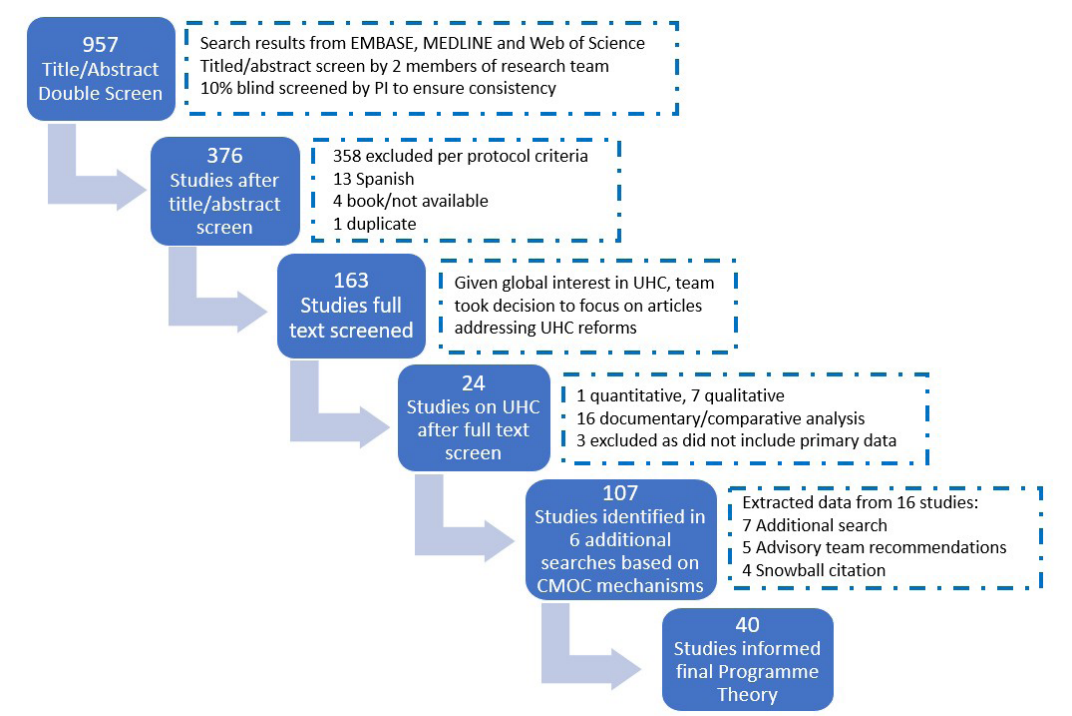
PRISMA Diagram for Formal and Additional Searches. Abbreviations: PRISMA, Preferred Reporting Items for Systematic Reviews and Meta-Analyses; CMOC, context-mechanism-outcome configuration; UHC, universal health coverage; PI, principal investigator.

 In line with realist practice,^17^ an additional targeted, theory-driven search was conducted to assist in the refinement of the CMOCs and the development of our finalised Programme Theory. This search included literature on UHC and theories on coherence, cohesion, collaboration, distributed leadership, fragmentation and trust. The search resulted in 988 articles. Of these, 107 were deemed somewhat related to our research, with seven found to enhance the development of the final Programme Theory. Additionally, five articles recommended by our research advisory team informed the development of our refined CMOCS and Programme Theory. Snowballing or citation tracking from these additional studies yielded four further articles for inclusion. Ultimately, these 16 additional articles assisted further in the development of our final Programme Theory. These 16 articles, coupled with the 24 articles from the initial or “main” literature search, meant that a total of 40 articles were included in this review, and informed our final Programme Theory. See Figure 2 for a PRISMA (Preferred Reporting Items for Systematic reviews and Meta-analyses) diagram of the number of articles included, as well as Supplementary file 1 for a listing of the articles included in the additional search.

###  Stage 6: Development of Refined CMOCs and Final Programme Theory

 Through iterative interrogation of the data, integration of the literature from the additional search and discussions with our research advisory team, we developed our refined CMOCs and our final Programme Theory. LC provided advice on realist methods and worked with LF to refine the CMOCs further. The preliminary CMOC analysis was presented to the stakeholder group in February 2024, to confirm, refine or refute the middle-range theories. SVB then assisted in the refinement of the CMOCs and helped to situate them within the international health landscape.

## Results

 Of the 24 studies included from the initial systematic search, 7 discussed UHC reforms in Latin America, 7 across Europe, 3 from Africa, 2 from Asia (both China), 1 from South-East Asia (India), and 1 from the Middle East (Iran). Three other articles compared health systems across continents, one comparing reforms across several LMICs, one comparing several middle and high-income countries and one comparing reforms across the five “BRICS” countries (Brazil, the Russian Federation, India, China, and South Africa). Of these studies, one was quantitative, seven were qualitative, and the remaining 16 were reviews, including documentary and comparative analyses.

###  Refined Context-Mechanism-Outcome Configurations

 Our analysis led to five themes including strong governance, financing, health system structure, political commitment and communication/relationships. The 11 CMOCs within these categories of interest are presented in Box 1, along with references to the literature that informed each CMOC. In line with realist methods, providing the specific references gives a sense of the weight of evidence supporting such theories or concepts. As noted above, some of the studies referenced are reviews across regions and therefore include the experiences of multiple countries. Please see Supplementary file 1 for details of the studies included in this review. A small number of examples from the data that informed the CMOCs are presented in Box 2. Please see Supplementary file 2 for further examples of data that have informed the development of the CMOCs.

**Box 1. Context-Mechanism-Outcome Configurations**

**Theme 1: Strong Governance**

CMOC 1: When service provision is decentralised without adequate funding or sufficient devolution of power^12^ or in countries where capacity and wealth vary significantly between regions,^22^ a lack of budget control and limited decision-making power at a local level^23,24^ or a lack of integrated planning between national and local authorities^23^ can lead to fragmented service provision and financial pathways,^23^ and a lack of coverage, particularly for marginalised groups.^22^

CMOC 2: When reforms do not address legacy issues such as resource constraints or weak infrastructure, when they are implemented through a top-down, centralised approach^25,26^ or when centralised management is out of touch with local needs,^27^ a lack of participatory, local decision-making and buy-in for reforms can act as a barrier to implementation.^27^

CMOC 3: When civil society and community organisations are involved in decision-making about healthcare and local services have the resources and authority to deliver reforms^22,25^ then decentralisation or a devolution of centralised power structures can strengthen social participation^22,23^ and thus facilitate UHC reforms.

**Theme 2: Financing**

CMOC 4: If financing pathways are overly complex or fragmented^23,28,29^ or if there exists a lack of adequate revenue^23,30^ or inequitable mechanisms for revenue collection^22,23,30,31^ then services are fragmented^29^ and a disparity between policy commitments and service delivery is created^27,32^ because technical design of funding mechanisms remains challenging and optimal financing mechanisms for reforms remain unclear.

CMOC 5: Sub-optimal pooling and purchasing arrangements^23^ and inadequate provider incentives^33^ influence provider behaviour in treatment decisions.^33^ These decisions in turn affect equity in access and can lead to the introduction of out-of-pocket payments for service users,^22^ a denial or rationing of services and longer wait times.^33^

CMOC 6: On the service user end, when designing and implementing UHC policy, premiums must be made affordable and there must be an equitable distribution of entitlements.^33^ Otherwise, there is unequitable access and a lack of financial protection/continuation of OOP payments,^22,26,28,33^ particularly for non-urban and marginalised groups.^28,33^

**Theme 3: Health Systems Structure**

CMOC 7: In countries whose public health systems are under-resourced or fragmented,^22,34,35^ a lack of resources or capacity^23,33^ means that the system is unable to deliver the breadth of services outlined in the UHC policy,^26,28^ creating a policy implementation gap^32,33^ and often forcing people to seek care from the private system.^24,26,35^ Because of this, access to public services can be constrained,^28,36^ financial protection will be limited,^22,26,37^ and the private sector will be strengthened.^24,26,28^

CMOC 8: For countries that want to introduce UHC, development of a strong primary health system^38,39,40^ can be used as a vehicle for achieving UHC.^22^

**Theme 4: Political Commitment**

CMOC 9: Political context significantly affects the trajectory of reforms, supporting or undermining implementation of UHC.^12,24,25,28,37^ The presence of a strong social movement that advocates for broad reforms^22,25^ and a multipartisan political consensus that supports and/or political commitment to the underlying values of UHC^12,25,41^ can facilitate sustained support of UHC reforms across the civil service and political parties and protect the reforms beyond election cycles.

CMOC 10: In a social context in which healthcare is valued as a human right or public good and not conceptualised as a commodity or linked to capacity to pay/employment status,^13,22,24,25^ enshrining universalism within a legal/constitutional framework^25,28,36,37^ can provide a foundation that helps to hold governments accountable and protect reforms against the changing political agendas.^13,24,28^

**Theme 5: Communication and Relationships**

CMOC 11: When a government is committed to implementing health reforms, a clear narrative around what the reforms involve,^31,38,42^ meaningful engagement with the public and stakeholders working in the public health system^12,25,29,38^—including clear communication with the population and frontline workers about what the reforms mean for them^26,33,38,39,43^—can contribute to the advancement of reforms.^25,29^

---------------

Abbreviations: UHC, universal health coverage; CMOC, context-mechanism-outcome configuration; OOP, out-of-pocket.

**Box 2. Data Supporting Refined Context-Mechanism-Outcome Configurations**

**Theme 1: Strong Governance**

“Decentralisation has … generated more complex environments for governance and performance management, because of the varying capacity and wealth of different localities. If not effectively managed, decentralisation could further fragment decision making [and] widen inequalities between municipalities”^22^ (CMOC 1).

“In Indonesia, decentralization has strained the limited capacity of local governments to do integrated health planning and budgeting”^23^ (CMOC 1).

“Decision making was perceived to be top-down without community or health staff involvement”^26^ (CMOC 2).

“The centralisation reform …[reduced] opportunities for participatory decision making, … polarising finance and clinical managers. This not only hindered reform implementation, but also impacted negatively on the overall functioning of the health system”^27^ (CMOC 2).

“Decentralisation brought decision making and services closer to the users, especially for rural populations, and established a voice for civil society and a crucial platform for democratisation of health by empowering communities and increasing involvement of civil society and community organisations in decisions relating to health”^22^ (CMOC 3).

“In Brazil, Colombia, Peru, Uruguay and Venezuela, civil society provided the impetus for decentralisation, which was also used as a mechanism to deepen democratisation and citizenship by strengthening social participation”^22^ (CMOC 3).

**Theme 2: Financing UHC**

“Adequate financing is a major constraint for making progress towards UHC … which is sometimes aggravated by sub-optimal pooling and strategic purchasing arrangements and institutions”^23^ (CMOC 4 and 5).

“Achievement of UHC has been hampered by inequitable health financing and employment-based social insurance schemes, which have created parallel schemes and segmented the population”^22^ (CMOC 4 and 5).

“Out-of-pocket expenditure existed, despite the abolition of fees, when patients were forced to use private services or purchase drugs when they were unavailable in the public system”^26^ (CMOC 5 and 6).

“The new provider payments did not incentivize equity, efficiency and quality healthcare service provision due to perceived inadequacy in payment rates”^33^ (CMOC 5 and 6).

**Theme 3: Structure and Infrastructure**

“Reforms of benefit packages should also inform infrastructure developments, failure to which makes the benefit package merely a wish list, with limited access to actual services and limited financial risk protection”^33^ (CMOC 7).

“Problems with quality and waiting times for health services have forced [all segments of the population] to pay out of pocket to access healthcare”^22^ (CMOC 7).

“The dissatisfaction of Italians with respect to the efficiency and quality of their healthcare ranks the highest in Europe. Largely as a consequence of this dissatisfaction, recourse to the private market for services has increased steadily in the last few years”^24^ (CMOC 7).

“Countries must build robust healthcare systems founded on PHC to ensure access to quality preventative and curative healthcare”^38^ (CMOC 8).

**Theme 4: Political Commitment**

“Reforms to strengthen health systems and achieve universal access to healthcare should be cognizant of the importance of the socio-political context … That context determines the nature and trajectory of reforms promoting universality or any pro-equity change”^25^ (CMOC 9).

“Implementation of health financing reforms for UHC is inherently political”^42^ (CMOC 9).

“Healthcare systems that aim to achieve universality or bring about sustainable pro-equity change cannot do so unless the broader socio-political context is conducive to such a change”^25^ (CMOC 9)

“Legislation can preserve key reform components through future political fluctuations”^12^ (CMOC 10).

“All Southern European countries have recently passed legal reforms in order to turn their (public) social insurance systems into national healthcare systems. The shift from a social insurance healthcare system to a national health service entails two basic conditions: coverage of the whole population as a citizenship right (as opposed to workers and their dependants in social insurance systems), and the deﬁnition of a comprehensive package of services for all users, independently from occupational status”^36^ (CMOC 10).

“Chile, Colombia, and Mexico introduced organisational changes that emphasised the intrinsic value of health for citizenship”^22^ (CMOC 10).

**Theme 5: Communication and Relationships**

“Setting a clear, compelling vision enabled leaders to mobilise stakeholder commitment”^38^ (CMOC 11).

“While other Latin American countries implemented health reforms solely in a top-down direction, Costa Rica’s strategy of deep community engagement strengthened their reform by creating transparency and building buy-in”^38^ (CMOC 11).

“China’s effort to engage the domestic academic and research community, and international agencies, has generated a strong evidence and technical basis on which the government has built China’s health system reform”^29^ (CMOC 11).

“The lack of clarity on the mechanism and route to achieve UHI also meant that it failed to gain public or political support”^31^ (CMOC 11).

“The government’s responsibility to properly and continuously inform all citizens about their reforms cannot be overemphasized. Some of the confusion expressed could probably have been avoided by better coordination and communication from those responsible for implementation of the reform”^26^ (CMOC 11).

---------------

Abbreviations: PHC, primary healthcare; UHC, universal health coverage; CMOC, context-mechanism-outcome configuration; UHI, universal health insurance.

###  Refined CMOCs for Theme 1: Strong Governance

 Health system reforms often incorporate significant institutional and organisational changes.^12^ Within this, decentralisation is a common component of UHC reform and the studies within this review shed light on this widely discussed issue.^30,36^ As the data from this review indicates, a lack of budget control and decision-making authority at a local level, as well as a lack of integrated planning between national and local authorities, can lead to fragmented services and financial pathways, particularly for marginalised or vulnerable communities. This in turn undermines progress towards UHC. Decentralisation also became problematic in relation to access and equity when there were significant variations in wealth and health system capacity between regions or states within a country.^22^

 While the data warns against the pitfalls of ill-planned out decentralisation, countries have also found that when UHC reforms are implemented through a top-down, centralised approach, or particularly when central management are out of touch with local needs, a lack of local buy-in and participatory decision-making can act as a barrier to reform implementation.

 When governed or managed well, decentralisation can empower local services and organisations and can facilitate the successful implementation of reforms. In particular, when civil society is involved in decision-making around healthcare and when local services have the authority and resources to deliver reforms, UHC reform implementation is facilitated. This can be seen, for example, in Brazil.^25^

 Thus, these international experiences highlight the significant role of governance within UHC reforms. As further indicated by the data, governance structures shape health financing, which will be discussed below.

###  Refined CMOCs for Theme 2: Financing UHC

 For countries implementing UHC reforms, the challenge for political and health system leaders becomes how to raise and sustain funding for the ongoing delivery of services, while also financing increased coverage of services and financial protection as outlined in their proposed UHC reforms. For countries whose health sectors are chronically underfunded, this is particularly challenging, while countries that have large informal employment sectors face additional complexities when attempting to finance UHC reforms.

 Health financing resources the interactions between service providers and the public and defines who pays for care, when they pay, how much they pay, who they pay and obtain services from, and what types of services they can receive.^45^ Thus, fragmentation or a lack of coherence across financing has a significant impact on health equity and accountability, and thus on the goals of UHC reforms.^22,33^ This fragmentation can occur across all elements of health financing, including revenue raising, the pooling of funds and the purchasing of services, with consequences for the provision of services.^46^

 Furthermore, ensuring that reform policy and implementation gives sufficient consideration to provider reimbursements, service user premiums and the interaction between the two is integral if the UHC goals of equitable access and financial protection are to be realised. Without this strategic planning, countries can be faced with a workforce that is not paid in a timely manner, which can then lead to providers rationing their services or treatments.^33^ This can lead to restricted access to the public system for service users, who in turn must pay out-of-pocket (OOP) to receive timely treatment or, if they can afford to, turn to private health insurance (PHI).^26^

 Finally, political commitment to financing the health system, and UHC reforms in particular, significantly impact implementation. However, this will be discussed further below in the section on politics.

 Thus, the data suggests that the financing of UHC is a highly complex issue, with the country-specific context significantly influencing the mechanisms that trigger outcomes throughout the policy implementation process. However, if a country is to achieve policy implementation, the health system must have the capacity to deliver on UHC policy promises. The link between health financing, health systems and UHC reforms will be discussed below.

###  Refined CMOCs for Theme 3: Health System Structure and Infrastructure

 A crucial factor determining reform policy adoption is the character of the established health system,^36^ with those that are well-functioning most capable of supporting UHC implementation.^22,47^ A number of countries within this review found that a lack of capacity within their public health systems acted as a barrier to implementing UHC reforms, including access and financial protection. This lack of capacity was often the result of a lack of financial resources due to historic underfunding of the health system, poor national economies and/or colonial legacies.^23,48,49^ For some countries, austerity measures taken in response to the economic crisis of 2008 resulted in a health system that was significantly underfunded.^31^ This lack of financial resources in turn led to workforce shortages, a lack of healthcare facilities and services and the unavailability of medicines.

 Fragmentation within and across health systems also played a key role in diminishing the capacity of a health system to implement UHC reforms. For many Latin American countries, fragmentation of organisation and services posed a barrier to health system reform.^22^ In the BRICS countries, fragmentation occurred whennational policies had to be implemented by subnational entities that were largely autonomous.^35^ These issues often occurred in tandem with the fragmented financial pathways outlined in the previous section, and were often caused or acerbated by these resource constraints.

 This lack of capacity, resources and fragmentation within the public system formed barriers to accessing care and increased OOP payments. These issues meant that many members of the public turned to the private sector for care, either because they could afford to do so or because they felt they had to in order to receive care in a timely manner.^24,26^

 However, while many of the included studies discuss policy implementation gaps and barriers to access due to capacity inefficiencies, other studies, particularly those examining the Costa Rican experience, offer a roadmap for strengthening capacity on the journey towards UHC. In Costa Rica, health system reforms were facilitated through the development of their primary care system through the use of integrated public health services and primary care delivery, multidisciplinary teams, geographic empanelment and reliable data and monitoring systems.^39^

 While strong governance and financing underpin health systems and determine system structure and service delivery, it is also political commitment that facilitates UHC reform implementation.

###  Refined CMOCs for Theme 4: Political Commitment Underpinned by Social Movements

 Political and social issues have significantly impacted the shape of health systems^12,25^ and change brought on by collective action within these arenas has often been the impetus for health reforms.^22^ Our data suggests that lack of political commitment to health reforms posed a significant barrier to implementation.^25^ This lack of political commitment can stem from political concerns (often from the Ministry of Finance) over the financing of the reforms, the ideologies of the particular political parties or personalities in power during the reforms^33,41^ or broader socio-political contexts, particularly ones in which conservative and/or neoliberal ideologies prevail.^25,41^

 In many Latin American countries, pro-equity social movements underpinned UHC reforms. These social movements often emerged as reaction to neoliberal ideologies and reforms, which had exacerbated social inequalities.^25^ With the exception of Cuba, these neoliberal ideologies, often introduced by political leaders or through economic policy reforms and conditions imposed by international financial institutions,^22^ also bled into the health systems, further exacerbating inequalities. A consequence of these class-based disparities was a decline of population health, especially for those within marginalised groups.^25^ Thus, while the social justice movements in the region—which often rose as a response to these inequalities—helped bring about UHC reforms and improve population health, countries also had to contend with oppositional neoliberal ideologies that emphasised efficiency and personal responsibility over social protection and responsibility.

 However, strong political support for reforms—often, but not always, from left-leaning parties—facilitated the introduction and adoption of reforms. Within the research included in this review, this support was often situated within broader social movements committed to equity, justice, human rights, and social responsibility.^22,25^ Another factor that facilitated reforms was this protection of healthcare as a human right within a constitutional or legislative framework.^22,24,36^ This protected health reforms against the changing agendas of politicians, while also protecting individuals’ right to health, independent of their citizenship or employment status.

 Integral to political support, as well as engagement among actors across the public health system, is communication and collaboration between stakeholders.

###  Refined CMOC for Theme 5: Communication and Relationships

 Across the studies included in this review, communication and engagement with and between key stakeholders appears to facilitate the successful implementation of UHC reforms. Specifically, these stakeholders include politicians, policy-makers, health system managers (national and local; acute and primary sectors), the healthcare workforce, members of the public (including community and marginalised groups) and researchers. Across this matrix of relationships, fragmentation can occur on a number of levels.

 In turn, communication and engagement are closely aligned with and supported by robust governance. If stakeholders across the health system engage meaningfully with each other in decision-making and communicate effectively, then health reforms are facilitated. This is particularly true when decentralisation is included within the reforms. If joined-up decision-making is not present, fragmentation is created or exacerbated, and centrifugal forces gain the upper hand.

 We have presented our CMOCs and some of the data that informed them. We will now discuss the causal links between the CMOCs and situate them within our Programme Theory.

###  Programme Theory

 The Programme Theory synthesising the full set of findings from this review suggests that a set of interlinking factors working in tandem facilitates the successful implementation of UHC reforms. In particular, cohesion and coherence across the political context, the functions of governance and financing, across the health system structure and within stakeholder relationships supports successful implementation. Attention must thus be paid to a holistic understanding of health systems and the country contexts of UHC reforms.^32^ This understanding can underpin cohesion across these five facets, helping health system leaders tailor UHC reforms to their unique country context. In contrast, fragmentation across these sectors and functions forms a barrier to the implementation of UHC. Understanding these relationships enables health system leaders to understand how the aspects that each CMOC highlights relates to the health system as a whole. Thus, although these themes have been presented within separate CMOCs, in order to highlight the causal relationships within each of them, it is important to bear in mind that they are inextricably interlinked, being capable of acting as a support or hinderance for the other components. Thus, the international experiences in this review can act both as a warning and as a roadmap for other countries attempting to implement UHC.

 According to Becerril-Montekio et al,^44^ fragmentation can occur across institutions, health financing, healthcare levels, governance, and organisational models within a health system. Fragmentation leads to inefficiencies, the persistence of inequities, a propensity for catastrophic health expenditure and, in general, acts as a barrier to providing effective UHC. Furthermore, they suggest that segmentation refers to the separation of the population according to their position within the labour market. This segmentation leads to a categorisation of population groups and decisions around who deserves greater access and services, and can often act as a barrier to access and universalism. This issue arises within our CMOCs around governance and health system structures, with populations segmented in accordance with their employment status or age, for example, exacerbating inequities. Thus, the avoidance of both segmentation and fragmentation is an important consideration in relation to our findings around coverage and in relation to a health system’s capacity to deliver comprehensive, integrated care.

**Figure 3:**
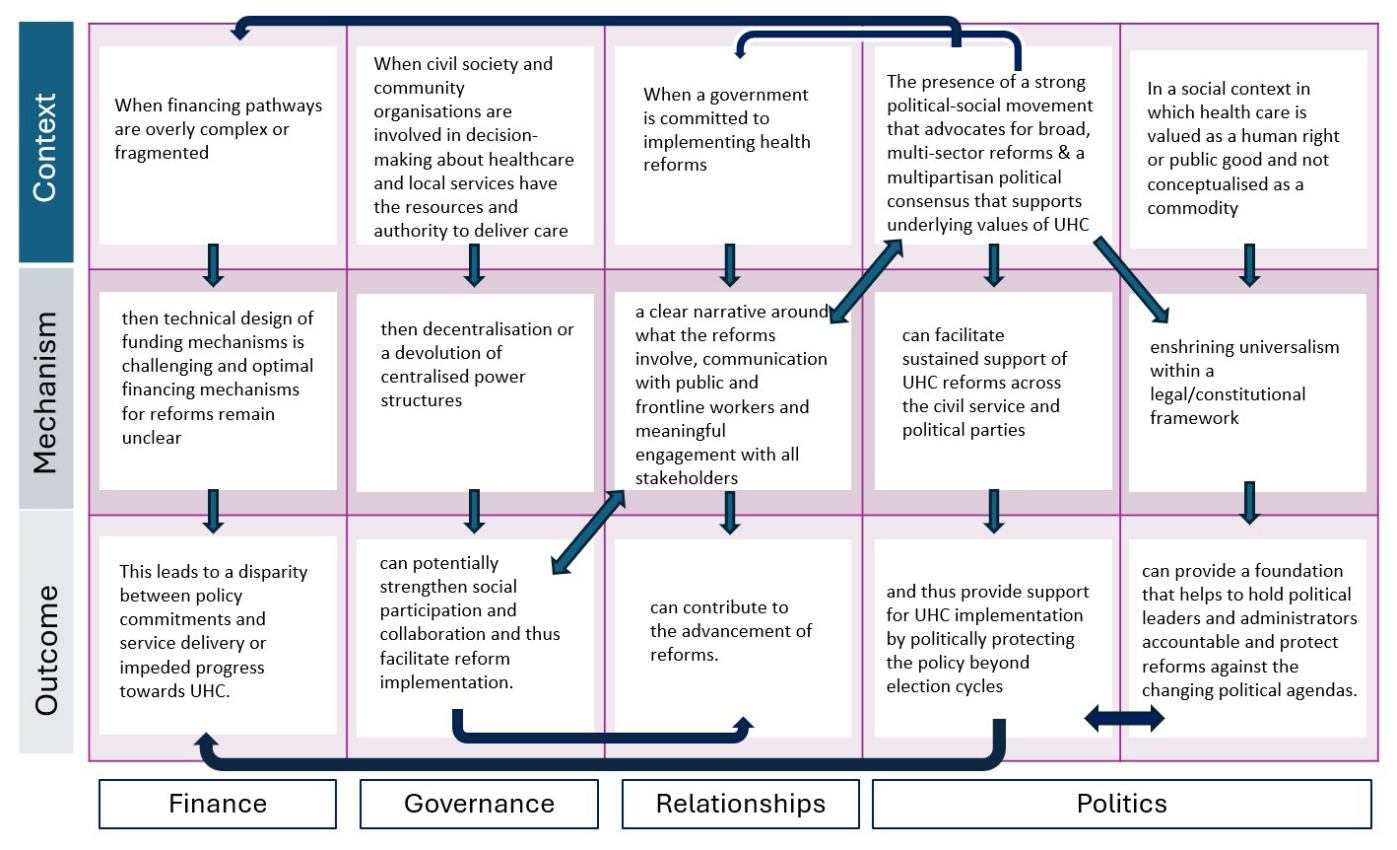
Context-Mechanism-Outcome Configurations and illustration of causalities. Abbreviation: UHC, universal health coverage.

**Figure 4:**
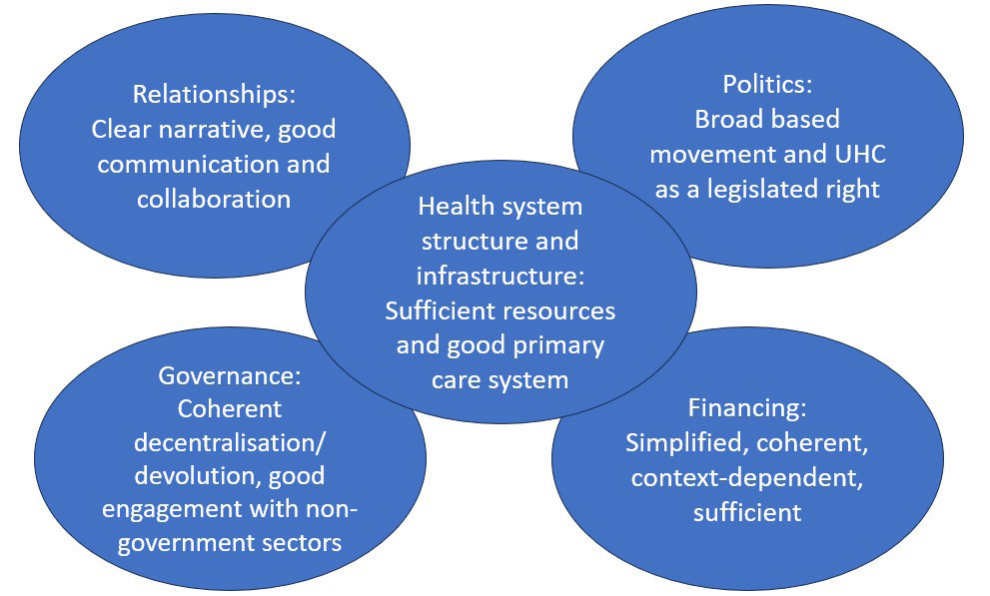
Findings and Knowledge Contribution Within the Themes. Abbreviation: UHC, universal health coverage.

**Figure 5:**
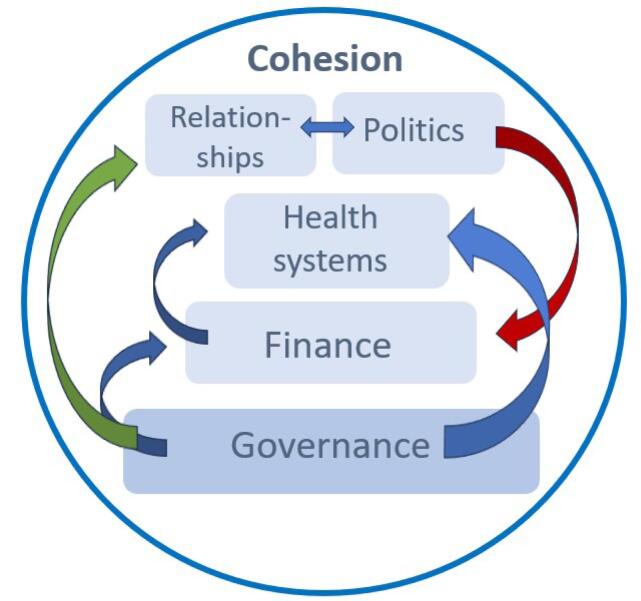
Programme Theory – Themes and Relationships.

 We suggest that in contrast to fragmentation and segmentation, cohesion between health system functions and sectors can support countries to implement UHC. Some of the causalities between finance, governance, relationships, and politics are illustrated in Figure 3, through abbreviated CMOCs. For example, political commitment supports the extent of finances earmarked for health, while communication between policy-makers, members of the public and frontline workers supports collaboration. Figure 4 illustrates the distillation of our analysis in the form of our main findings within the five themes. The roles and relationships between these five themes are illustrated by Figure 5. Within this Programme Theory, strong governance underpins the four other aspects of a health system. Building out of this must be the financial means and appropriate funding mechanisms for both the health system and reforms. Politicians influence how much of the national budget is earmarked for health, and health systems rely heavily on a political commitment to maintain service delivery while also financing reforms. Furthermore, financing supports the functioning of the health system itself, impacting infrastructure, workforce and service delivery. The shape of a health system determines the relationships among stakeholders. The strength of engagement and communication between these stakeholders and politicians then determines the success of UHC reform implementation. In order to function well and for UHC implementation to be facilitated, all these aspects of the health system must work in cohesion with each other.

 The theory that cohesion between these areas facilitates the successful implementation of UHC is echoed in the international literature. Chuma and Okungu,^50^ for example, suggest that a systemic or harmonised approach to health financing reforms is necessary in order to achieve UHC, and to prevent fragmentation and segmentation. Domapielle,^51^ meanwhile, suggests that the success of UHC implementation hinges on three factors: a strong political commitment to the objectives of UHC, a robust economy that includes a broad tax base and the capacity to adequately mobilise taxes, and a health system that has the capacity to deliver UHC. Furthermore, Uzwiak and Curran^52^ insist that community participation in healthcare must have multisectoral support from the government in order to be meaningful. These arguments are closely aligned with our own findings.

 Speaking more broadly, Becerril-Montekio et al^44^ state that health systems exist among and interact with other social systems. This is echoed by Bertram and Koonin,^53^ who suggest that social protection interventions should be a core part of UHC implementation, as they recognise the social determinants of health. Furthermore, Thomas and Fleming et al^54^ suggest that health system resilience is best understood as a nested concept – nested within and related to the health of communities, cities and society as a whole.

 Thus, in contrast to fragmentation, a cohesive approach among all the health system functions and other sectors within society facilitates UHC reforms. Specifically, the factors that facilitate or impede reform have been articulated through our themes and CMOCs. These will be discussed in detail below.

## Discussion

 Domapielle^51^ suggests that the journey towards UHC is not a “one size fits all” process but instead a long-term policy engagement that requires adaptation to each country’s unique socio-political, cultural and economic context. This is reminiscent of Wilsford, who suggests that bringing about big changes often requires “assiduous cultivation of the policy soil”^55^ (p. 277). Within this context, our findings in regard to governance and the key factors that enable successful decentralisation; in relation to determining financing mechanisms for UHC that are context specific; and in relation to building public health system capacity so as to facilitate UHC reforms all contribute new insights to international discourses on UHC. Furthermore, our findings that political commitment to UHC, which can include a legal framework enshrining health as a human right, can be facilitated within the context of a broader social movement, as well as strong engagement among public health system stakeholders, also offer additional nuances to the international discourses regarding UHC reforms. These ideas will be discussed below within the context of our themes.

###  Robust Governance

 Effective governance is central to the successful functioning of health systems and inextricably linked to health system reform,^43^ playing an essential role in the introduction of UHC.^56^ Decentralisation, a significant feature of many UHC reforms, is often conceptualised as a management strategy, a top-down intervention from a central government to lower levels of government.^57^ However, internationally, the results of decentralisation are largely mixed, achieving varied levels of success in relation to their intended effects on equity in population health outcomes and health system efficiency.^58^ Our review indicates that decentralisation, if not underpinned by strong governance structures and integrated decision-making, can act as a barrier to reform and not lead to greater equity and access. However, when done well in favourable contexts, decentralisation can lead to improved access, utilisation and service delivery,^59^ to stronger civic engagement, community empowerment and a democratisation of health.^22^

 Along with examining the contexts and mechanisms surrounding decentralisation that support reform implementation, it is interesting to consider the ways in which governance structures can support or impede change. Wilsford^55^ suggests that centralised, hierarchical health systems are often more successful at introducing big policy change and leveraging new policy paths than those that are decentralised and non-hierarchical. Therefore, each country must ask itself, giving its country-specific context, which type of reform—a “big bang” one, as seen recently in Finland,^60^ for example, or one that implements reforms through incremental steps—is more likely to be successful given its overall governance system.

###  Financing Reforms

 Financing is considered one of the most contentious elements of policy design for health reforms, as they directly influence who pays for and who benefits from the healthcare system.^61^ This makes it one of the most challenging aspects of UHC reform.^1^ However, it is also a critical element in determining the degree to which health systems accomplish redistribution, universality and risk protection,^1^ as the mechanisms through which healthcare is financed can mitigate or exacerbate inequalities.^62^

 The primary models for financing UHC are based on the Beveridge/Bevan National Health Service system financed through general taxation, and the Bismarckian, or Social Health Insurance (SHI), system based primarily on employer-based sickness funds. No matter the model under consideration, countries that have large informal employment sectors face additional challenges in relation to revenue collection. While the Beveridge model helps to ensure vertical equity in the pooling arrangements, it has been much more successful in HICs than in LMICs, where the tax base is often narrow, with limited capacity to mobilise funds for the health sector.^63^ However, SHI models in LMICs have been found to exclude informal sector workers and their families,^64^ leading to a lack of coverage for poorer populations.^65^ Furthermore, countries are more likely to progress towards UHC if they reduce OOPs in a progressive way through de-linking entitlement from payment of SHI contribution.^65^ This suggests that the SHI model is often sub-optimal for the successful implementation of UHC.

 It is also important to note that countries can utilise a pluralistic public financing framework when financing UHC. Furthermore, international donors and PHI schemes also operate in most countries around the world, although PHI reduces equity in health systems by removing well-off groups from pooling arrangements and by widening the disparities in care available to different population groups.^64,66^ HICs, and LMICs such as Costa Rica and Thailand, that have attained a reasonable semblance of UHC rely largely on public financing arrangements, rather than PHI.^32^ The relationship between the public and private sectors vis-à-vis UHC reforms will be discussed below, though it deserves a further exploration that is outside the remit of this study.

 The literature that informed our finance and health systems CMOCs suggest that along with collection, the pooling and purchasing functions must also be given adequate consideration if UHC is to be achieved. This is echoed by Chuma and Okungu,^50^ who suggest that the design of pooling and purchasing mechanisms can influence provider behaviour, and that these functions are central to the achievement of UHC. Economic and infrastructural realities are thus an additional, and significant, consideration for countries attempting to finance and meet the goals of UHC, and pragmatic financing approaches that are appropriate to a country’s context must be considered if implementation is to be successful.^51^

 The goal of UHC to reduce catastrophic health spending and eliminate financial barriers to accessing care stems in large part from the fact that OOP payments for healthcare results in drastic reductions in access to health services and increases catastrophic health expenditure for households.^67^ Thus, the work to determine appropriate financing mechanisms for UHC is not simply theoretical but has real-world implications for individual and population health outcomes, and for the ability of families to meet their daily needs.

###  Health System Structures and Infrastructure

 The data in relation to health system structures suggests a symbiotic relationship between UHC policy commitments on the one hand and health system capacity and infrastructure on the other. If a health system lacks the capacity and cohesion to deliver on UHC commitments, then there exists a policy implementation gap.^32^ However, a lack of capacity within the public health system—often manifesting in the form of long waiting times or lack of availability of services or medicines—can also drive many into the private sector. These ongoing contributions to the private system in turn can mean the private sector is bolstered at the expense of the public system, thus creating a barrier to UHC implementation.

 However, the research included in this review provides a roadmap for building capacity within the public system. Specifically, it provides further evidence to support the WHO position that strong PHC systems are both essential to and facilitate the establishment of UHC, supporting access and minimising financial hardship.

###  Political Commitment Underpinned by Social Movements

 Health reforms are inherently political, as they herald in change and reallocate resources amongst different actors.^61^ This review adds evidence to the literature that highlights the significant role that politics and the political context of a country plays in UHC reform. Witter et al, for example, suggest that in fragile states, the uptake of health system reform policy has been strongly driven by political considerations.^68^ While the processing of converting to a UHC system is fraught with political challenges, a better understanding of these challenges can assist in the implementation process.^1^ These political challenges can hail from myriad actors including opposition parties concerned reforms are going too far or not far enough, ministries of finance concerned about expenditure and other interest groups concerned about maintaining the status quo. However, we suggest that understanding potential solutions to these challenges is also essential to the facilitation of UHC implementation.

 Thus, our findings indicate that UHC reforms can be facilitated by political vision, conviction and commitment to UHC goals.^12,13^ While UHC reforms have been adopted without multipartisan political support,^69^ our findings indicate that such cross-party consensus significantly facilitates long-term political commitment to UHC implementation. This support has often emerged from socio-political contexts that were ripe for change, bringing in health reforms as part of larger civil rights reforms or driven by popular demands of newly enfranchised voters.^70^

 It is important to note that for some countries, health reforms were not driven by popular support but were instead elite-driven.^1^ This was the case in Mexico, for example, where reform was driven by the minister for health and a “change team” of technocrats who supported him.^70^ This experience was echoed in Taiwan and Korea, where reforms were largely politician-led.^71,72^ The experiences of these countries suggest that reforms can be created and passed into law by elite groups. However, in the case of Mexico, implementation of the reforms proved challenging, as they were not founded on popular support.^70^ Our data builds on this, suggesting that the implementation of UHC reforms is facilitated when they are situated within popular social-political reform movements.

 Furthermore, our findings suggest that this political commitment to UHC can be protected against changing political agendas by enshrining health as a human right within a legal framework,^13^ as was the case in Brazil, South Africa, and Spain.^28,35^ While individual politicians work within the liminal timeframe of election cycles, the benefits of implemented UHC reforms take many years to unfold.^1^ Multipartisan political support and the legal protection of health as a human right thus help to protect UHC reforms beyond election cycles.

###  Collaboration, Communication, and Engagement With Key Stakeholders

 The studies included in this review indicate that collaboration and communication between members of the public, civic organisations and those working within the public health system who are designated to deliver UHC reforms significantly facilitates the implementation of reforms. Communication fosters understanding about reform goals, the mechanisms and strategies for achieving them and the implications for service delivery and access. These discussions can then facilitate a collective commitment to the reforms and the necessary steps to implementation.

 These findings are mirrored in other international studies, with Chuma and Okungu^50^ finding that engagement with the public promotes buy-in and acceptability. Birn et al, meanwhile, write that participation of the public and healthcare workers in decision-making and management is crucial to achieving universalism.^73^ As seen during the COVID-19 pandemic, enhanced communication and engagement between health system stakeholders helped to significantly improved universal access and services in countries such as Ireland.^74,75^

 Mahmood and Muntaner^25^ highlight the importance of support from other stakeholders within civil society, while other literature suggests that local community engagement with health facilities can increase equity, access and community accountability.^76^ This is aligned with the suggestion that community participation and engagement has long been a fundamental principle of both UHC and, within that, PHC.^13^ Thus, collaboration is not simply practical, in that it is the best way to reach consensus and accomplish collective goals. It is also an ethical principle that has underlined UHC and PHC reforms historically.

 UHC often faces opposition from political parties, as mentioned above, but also from a number of other stakeholders or interest groups, including the private insurance sector, doctors or, at times, unions concerned about proposed pooling mechanisms with national health insurance models. This is because UHC, and the financial reforms inherent within it, involves the redistribution of resources and power.^1^ However, while we acknowledge the significant role and influence these interest groups play in UHC reforms, and the significant body of literature that discusses these issues, our study adds new knowledge to UHC discourses by providing evidence around the ways in which collaboration among key stakeholders, particularly those in the public health sector, can facilitate reform implementation. Through this collaboration, it is possible to neutralise some of the opposition to reforms, improve civic engagement and support buy-in.

## Conclusion and Policy Recommendations

 The ultimate aim of a realist project is to identify solutions to complex problems. These findings point to potential solutions for those designing and implementing UHC policies and programmes. In particular, we recommend:

- Ensuring that those charged with implementing reforms have the authority and resources (financial, human, etc) to do so.
- Supporting reform implementation at a local level through ongoing dialogues between central and local health system managers.
- Practicing shared decision-making across the public health system and civil society organisations to help ensure that health services meet the needs of local populations.
- Governments commit funds to build capacity and finance UHC across the entire implementation period.
- Developing mechanisms for revenue raising that take into account the nature of the labour market and the country context.
- De-linking entitlements from employment status.
- Ensuring that pooling and purchasing arrangements adequately address capacity restraints and provider concerns.
- Creating clear messages that enable the public to understand the reforms and how they will affect service delivery and payments.

In order for UHC to be successful, it is important for each country to consider its unique context and capacities, learn from other countries in similar situations and create realistic plans for implementation. Without this, UHC, even if supported politically, will fall at the first hurdle. This paper sheds light on the unique contexts that facilitate or inhibit reform implementation, as well as some of the mechanisms that can be harnessed over time to facilitate UHC reform implementation. It is hoped that in doing so policy-makers can take these learnings and use them in reform implementation and, ultimately, make healthcare more universal, accessible and equitable.

## Limitations

 Along with highlighting shared barriers and facilitators to reform, the studies included in this review also demonstrate that no two health systems are exactly alike. Therefore, the facilitators for reform, and the strategies for actor management within these, will have to be adapted to each country’s unique context. The role that human judgement played in the development of the CMOCs and the theory-building articles included in the review must be acknowledged. Finally, the role of the private sector in relation to UHC reforms was outside the remit of this study. This issue is of significance, however, when considering UHC reform implementation, as private actors can play a role in improving access and coverage.^77^ This issue merits further investigation elsewhere.

## Acknowledgements

 The authors are grateful to Prof. Sara Burke, Director of the Centre for Health Policy and Management, Trinity College Dublin; Prof. Susan Smith, School of Medicine, Trinity College Dublin and Prof. Emeritus Carolyn Tuohy, University of Toronto for their role on the research advisory committee for the RESTORE project.

## Ethical issues

 Not applicable.

## Conflicts of interest

 Authors declare that they have no conflicts of interest.

## Supplementary files


Supplementary file 1. Articles Included in Initial Systematic Review and in Additional Search.

Supplementary file 2. Additional Examples of Data Supporting the CMOCs.
