# Supplementary material for: Barriers and Facilitators to International Universal Health Coverage Reforms: A Realist Review
Source: Int J Health Policy Manag. 2025 May 19;14:8709. doi: 10.34172/ijhpm.8709 (PMC12257192; doi:10.34172/ijhpm.8709)
Supplement: Supplementary file 1 — Articles Included in Initial Systematic Review and in Additional Search. [file ijhpm-14-8709-s001.pdf]

Article title: Barriers and facilitators to international Universal Health Coverage reforms: A realist review

Journal name: International Journal of Health Policy and Management (IJHPM)

Authors' information: Liz Farsaci[1*], Padraic Fleming[1], Louise Caffrey[2], Sara van Belle[3], Catherine O'Donoghue[1], Arianna Almirall-Sanchez[1], David Mockler[4], Steve Thomas[5]

[1]Centre for Health Policy and Management, School of Medicine, Trinity College Dublin, Dublin, Ireland.

[2]School of Social Work and Social Policy, Trinity College Dublin, Dublin, Ireland.

[3]Institute of Tropical Medicine, Antwerp, Belgium.

[4]Assistant Librarian Reader Services, Trinity College Dublin, Dublin, Ireland.

[5]Edward Kennedy Professor of Health Policy and Management, Centre for Health Policy and Management, School of Medicine, Trinity College Dublin, Dublin, Ireland.

***Correspondence to:** Liz Farsaci; Email: farsacil@tcd.ie

Citation: Farsaci L, Fleming P, Caffrey L, et al. Barriers and facilitators to international universal health coverage reforms: a realist review. Int J Health Policy Manag. 2025;14:8709. doi:10.34172/ijhpm.8709

Supplementary File 1. Articles Included in Initial Systematic Review and in Additional Search Articles Included in Systematic Review

First Author	Title	Year Published	Countries included	Aim/Purpose of study
1. Atim	Health financing reforms for Universal Health Coverage in five emerging economies	2021	Ghana, India, Indonesia, Kenya, South Africa	To contribute to closing the gaps in knowledge by synthesising information along key themes from five case studies of middle-income countries with political commitment to and some progress toward UHC, but where UHC systems are still in the process of development and face specific challenges.
2. Atun	Health system reform and universal health coverage in Latin America	2015	Latin America	To examine how health reforms in Latin American countries developed a distinct approach to health system reforms, which combined demand side changes to alleviate poverty and comprehensive primary health care to extend service access.
3. Berman	Towards universal health coverage: governance and organisational change in ministries of health	2019	Chile, Poland, Thailand, Australia	An exploratory comparative case study of four upper middle-income and high-income countries that offers insights into how UHC reforms are likely to affect health governance and the organisational functioning of an MOH accustomed to controlling the financing and delivery of healthcare.

4. Burke	From universal health insurance to universal healthcare? The shifting health policy landscape in Ireland since the economic crisis	2016	Ireland	To assess the gap between policy intent and practice and the difficulties in implementing major health system reform in Ireland.
5. Burke	Building health system resilience through policy development in response to Covid-19 in Ireland: From shock to reform	2021	Ireland	To examine whether and how the Irish government's pandemic response contributed to health system reform and increased resilience, including delivering UHC.
6. Cabeides	Adopting and adapting managed competition: health care reform in Southern Europe	2001	Greece, Italy, Portugal, Spain	To study adoption and adaptation of health care reform measures in Southern European countries.
7. De Vos	Colombia and Cuba, contrasting models in Latin America's health sector reform	2006	Colombia and Cuba	To analyse health system reforms regarding privatisation and the separation of purchaser and provider functions through comparing the experiences of Colombia and Cuba.
8. Duran	Universal coverage challenges require health system approaches: the case of India	2014	India	To use the case of India to demonstrate that UHC is about not only health financing; personal and population services production issues, stewardship of the health system and generation of the necessary resources and inputs need to accompany the health financing proposals.

9. Eckhardt	Universal Health Coverage in Marginalized Populations: A Qualitative Evaluation of a Health Reform Implementation in Rural Ecuador	2019	Ecuador	To explore the perceived effects of the 2008 health reform implementation on rural primary health care services and financial access of the rural poor in Ecuador.
10. Ferrera	The Rise and Fall of Democratic Universalism: Health Care Reform in Italy, 1978-1994	1995	Italy	To analyse the implementation and reform integrity of health reforms in Italy.
11. Giovanella	Universal health system and universal health coverage: assumptions and strategies	2018	Germany, the United Kingdom and Spain	To analyse trends in the contemporary health system reforms in a context of heavy financial pressures stemming from the economic crisis (2008-date) in European countries with universal health systems and to discuss their immediate and potential consequences for universal coverage.
12. Khankeh	Three Decades of Healthcare System Reform in Iran from the Perspective of Universal Health Coverage: A Macro-Qualitative Study	2021	Iran	To assess the progress and outcomes of UHC reforms in Iran over the past three decades.
13. Mahmood	Politics, class actors, and health sector reform in Brazil and Venezuela	2013	Brazil and Venezuela	To analyse the socio-political context in Brazil and Venezuela and look at how the changing state-society relations resulted in health being constitutionally recognised as a social right.

14. Mbau	Examining purchasing reforms towards universal health coverage by the National Hospital Insurance Fund in Kenya	2020	Kenya	To examine the influence of the purchasing reforms on Kenyan National Hospital Insurance Fund's purchasing practices and the implications of this for strategic purchasing and health system goals of equity, efficiency and quality.
15. Mhazo	The political economy of health financing reforms in Zimbabwe: a scoping review	2022	Zimbabwe	To understand the interaction between political and economic aspects of health financing reforms in Zimbabwe since the country got its independence in 1980.
16. Pesec	Primary Health Care That Works: The Costa Rican Experience	2017	Costa Rica	To provide a case study of Costa Rica's innovative implementation of four critical service delivery reforms.
17. Petmesidou	'Southern-style' National Health Services? Recent Reforms and Trends in Spain and Greece	2008	Spain and Greece	To analyse recent changes in the Greek and Spanish national health services and assess how the period of austerity and further recovery during the 1990s and early 2000s impacted on them in terms of equity and efficiency.
18. Rao	Progress towards universal health coverage in BRICS: translating economic growth into better health	2014	Brazil, the Russian Federation, India, China and South Africa	To discuss three key aspects of UHC reforms in BRICS: the role of government in financing health, the underlying motivation

				behind the reforms and the value of the lessons learnt for non-BRICS countries.
19. Rosa	Universal health care for Colombians 10 years after Law 100: challenges and opportunities	2004	Colombia	To addresses four central aspects of the country's health care reform following the 1991 Constitution, which mandated that health is a right.
20. Schut	Health Care Reform in the Netherlands: Balancing Corporatism, Etatism, and Market Mechanisms	1995	The Netherlands	To examine the transformation of the Dutch health care system and the impetus behind the market-oriented health care reforms implemented since 1989.
21. Spigel	Implementing sustainable primary healthcare reforms: strategies from Costa Rica	2020	Costa Rica	To provide an in-depth analysis of Costa Rica's specific implementation strategies, which offer tangible lessons and examples for other countries as they navigate the work of strengthening Primary Healthcare.
22. Tang	Advancing universal coverage of healthcare in China: translating political will into policy and practice	2004	China	To introduce and discuss the context and process of China's current health system reform and analyse how political will in China has been translated into policy practice over a decade.
23. Wishnia	Impact of financial management centralisation in a health system under	2020	South Africa	To study the impact of centralising financial decision-making on the functioning of the South African health system.

	austerity: a qualitative study from South Africa			
24. Yip	10 years of health-care reform in China: progress and gaps in Universal Health Coverage	2019	China	To assess the progress and gaps in UHC in China 10 years after reforms were introduced.

Articles included in additional search

First author	Title	Year published	Contribution to theory building
Abimbola	The impacts of decentralization on health system equity, efficiency and resilience: a realist synthesis of the evidence	2019	Concepts of decentralisation and governance, and impact on equity and resilience
Averill	Universal Health Coverage: Why Health Insurance Schemes Are Leaving the Poor behind	2013	Assisted with understanding of health financing models, particularly in LMICs.
Becerril-Montekio	Segmentation and fragmentation of health systems and the quest for universal health coverage: conceptual clarifications from the Mexican case	2024	Conceptualisation around fragmentation and segmentation
Birn	What matters in health (care) universes: delusions, dilutions, and ways towards universal health justice	2019	Definitions and conceptualisations of Universal Health Coverage / Care
Brennan	The impact of decentralisation on health systems in fragile and post-conflict countries: a narrative synthesis of six case studies in the Indo-Pacific	2023	Concepts of decentralisation, particularly in LMICS
Chuma	Viewing the Kenyan health system through an equity lens: implications for universal coverage	2011	Deepened understanding of health financing and equity
Cookson	The inverse care law re-examined: A global perspective	2021	Conceptualisations around fragmentation and health inequality
Derkyi-Kwarteng	A Narrative Synthesis Review of Out-of-Pocket Payments for Health Services Under Insurance Regimes: A Policy	2021	Deepened understanding of policy intention and language around implementation gaps

	Implementation Gap Hindering Universal Health Coverage in Sub-Saharan Africa		
Domapielle	Adopting localised health financing models for universal health coverage in Low and middle-income countries: lessons from the National Health Insurance Scheme in Ghana	2021	Deepened understanding of financing mechanisms
Hammonds	The emergence of a global right to health norm – the unresolved case of universal access to quality emergency obstetric care	2014	Conceptualisation around health as a right and policy evolution
He	Towards Universal Health Coverage via Social Health Insurance in China: Systemic Fragmentation, Reform Imperatives, and Policy Alternatives	2016	Assisted in understanding of fragmentation and financing
McGuire	Very high and low residual spenders in private health insurance markets: Germany, the Netherlands and the US marketplaces	2021	Assisted in understanding of financing mechanisms and private health insurance
Mcintyre	Fiscal Space for Domestic Funding of Health and Other Social Services	2014	Deepened understanding of differing financial contexts and mechanisms in LMICs
Ranabhat	Universal health coverage evolution, ongoing trend, and future challenge: A conceptual and historical policy review	2023	Conceptualising and understanding UHC definitions and policy evolution
Uzviak	Gendering the burden of care: health reform and the paradox of community participation in Western Belize	2015	Conceptualising community participation and political commitment
Wilsford	Path Dependency, or Why History Makes It Difficult but Not Impossible to Reform Health Care Systems in a Big Way	1994	Concepts of path dependency and change
