# Supplementary material for: Barriers and Facilitators to International Universal Health Coverage Reforms: A Realist Review
Source: Int J Health Policy Manag. 2025 May 19;14:8709. doi: 10.34172/ijhpm.8709 (PMC12257192; doi:10.34172/ijhpm.8709)
Supplement: Supplementary file 2 — Additional Examples of Data Supporting the CMOCs. [file ijhpm-14-8709-s002.pdf]

Article title: Barriers and facilitators to international Universal Health Coverage reforms: A realist review
Journal name: International Journal of Health Policy and Management (IJHPM)
Authors' information: Liz Farsaci[1*], Padraic Fleming[1], Louise Caffrey[2], Sara van Belle[3], Catherine O'Donoghue[1], Arianna Almirall-Sanchez[1], David Mockler[4], Steve Thomas[5]
[1]Centre for Health Policy and Management, School of Medicine, Trinity College Dublin, Dublin, Ireland.
[2]School of Social Work and Social Policy, Trinity College Dublin, Dublin, Ireland.
[3]Institute of Tropical Medicine, Antwerp, Belgium.
[4]Assistant Librarian Reader Services, Trinity College Dublin, Dublin, Ireland.
[5]Edward Kennedy Professor of Health Policy and Management, Centre for Health Policy and Management, School of Medicine, Trinity College Dublin, Dublin, Ireland.
***Correspondence to:** Liz Farsaci; Email: farsacil@tcd.ie
Citation: Farsaci L, Fleming P, Caffrey L, et al. Barriers and facilitators to international universal health coverage reforms: a realist review. Int J Health Policy Manag. 2025;14:8709. doi:10.34172/ijhpm.8709

Supplementary file 2. Additional Examples of Data Supporting the CMOCS

Strong Governance:

> *"Health system reforms often formally incorporate significant institutional and organisational changes such as the movement of financing to a third-party payer or decentralising authorities for healthcare delivery to local governments or individual facilities. However, our case studies suggest that an MOH can retain substantial power and influence in financing and delivery even when formal reforms remove these powers."*[12]
>
> *"In the highlighted countries, institution*-based *norms of government power and authority continue to influence de facto governance relationships in the health sector even when de jure organisation restructuring occurs to the MOH and/or a health financing agency."*[12]
>
> *"In Brazil, Colombia, Peru, Uruguay, and Venezuela, civil society provided the impetus for decentralisation, which was also used as a mechanism to deepen democratisation and citizenship by strengthening social participation."*[22]
>
> *"Decentralisation brought decision making and services closer to the users, especially for rural populations, and established a voice for civil society and a crucial platform for democratisation of health by empowering communities and increasing involvement of civil society and community organisations in decisions relating to health."*[22]
>
> *"However, decentralisation also generated more complex environments for governance and performance management, because of varying capacity and wealth of different localities."*[22]

"Decentralisation has improved citizen participation in health systems, but has also generated more complex environments for governance and performance management, because of the varying capacity and wealth of different localities. If not effectively managed, decentralisation could further fragment decision making, widen inequalities between municipalities, politicisation of health decisions."[22]

"In Latin America, effective regulation of health insurers and providers in public and private sectors has been challenging. Private insurers practise so-called cream skimming by enrolling low-risk high-income population segments, with adverse effects on equity, cost, service quality, and appropriateness in Argentina, Brazil, Chile, Colombia, Mexico, and Peru. Regulation of public insurers and providers has been hampered by bureaucracy and rigid public sector laws that have hindered effective management and competition."[22]

"Most of these five countries have complex decentralised systems and the variability across decentralised states or provinces considerably affects UHC reforms. These include powerful states in India, provincial governments in South Africa and counties in Kenya."[23]

"In Indonesia, decentralization has strained the limited capacity of local governments to do integrated health planning and budgeting, compounded by multiple and fragmented financing lines and poor data quality and use. While the bulk of government health expenditure occurs at the district level, the central government remains the dominant source of revenues. Complex and fragmented inter-fiscal government transfers in a decentralized system resulted in wide variations of health spending across districts. Also, these transfers are not performance-oriented to influence districts to allocate resources to achieve better health system results. District health offices rarely plan the use of this funding in a holistic manner to address gaps in inputs for health services, focused on populations and diseases of greatest need."[23]

"Four countries in this group have complex decentralised systems, such as counties in Kenya, provinces in Indonesia and South Africa, and states in India, which can be a source of strength if these encourage local ownership, commitment, and participation, but can also make reform slower to achieve given variability across multiple decentralised units."[23]

"Decision making was perceived to be top-down without community or health staff involvement, which also has been reported from reforms elsewhere."[26]

"While many managers expressed their loyalty to the organisation and how this had kept them motivated, the authoritarian management style, and the impact of centralisation on service delivery, had negatively affected organisational planning and decision-making."[27]

"Many managers in our study reported that the head office was disconnected from the rest of the health system, making them ill-suited for centralised control. However, given the fear of uncertainty, finance managers remained wary of financial decentralisation, as is common during fiscally constrained periods, and therefore, the reform has remained in place."[27]

"The PDoHs centralisation reform influenced its OC, reducing opportunities for participatory decision making and polarising finance and clinical managers. This not only hindered reform implementation, but also impacted negatively on the overall functioning of the health system."[27]

"The third [major problem] is the implementation of national plans based on the proposal by international agencies or the experiences of other countries without any localisation of the plan."[34]

"Governance restructuring required clarifying the MOH and CCSS's relationships and the redistribution of human and financial resources."[38]

Financing UHC:

"With the exception of Brazil, Cuba, and Costa Rica, achievement of universal health coverage has been hampered by inequitable health financing and employment-based social insurance schemes, which have created parallel schemes and segmented the population into three categories: (1) the poor, unemployed, and employed without social security; (2) the salaried working population with social security; and (3) the rich with private insurance."[22]

"Problems with quality and waiting times for health services has forced all three groups to pay out of pocket to access health care."[22]

"In Indonesia, decentralisation has strained the limited capacity of local governments to do integrated health planning and budgeting, compounded by multiple and fragmented financing lines and poor data quality and use. While the bulk of government health expenditure occurs at the district level, the central government remains the dominant source of revenues. Complex and fragmented inter-fiscal government transfers in a decentralized system resulted in wide variations of health spending across districts."[23]

"In several of the cases, inadequate public funding for health remains a key issue, for example in Ghana (despite dedicated sources of taxation, suggesting that ringfenced revenue for health is by no means a panacea for improving funding levels), India and Indonesia. In some cases, low levels of funding result both from weak government systems of taxation and lower prioritisation of health in the budget."[23]

"Adequate financing is a major constraint for making progress towards UHC in most of these countries, which is sometimes aggravated by sub-optimal pooling and strategic purchasing arrangements and institutions."[23]

"The PDoH has experienced a proliferation of 'unfunded mandates' in the pursuit of … As a result, recent service delivery plans have become divorced from the available funding…"[27]

"Reform implementation is constrained by financial resource mobilization and motivation, especially at provincial, municipal and county levels, in a highly decentralized environment. In particular, aligning resource allocation and incentives at these levels with the national priorities and health system reform objectives is an on-going challenge."[29]

"Sub-national governments, however, are neither explicitly accountable nor necessarily in a sufficiently strong fiscal position to comply with the national policies. Whereas provincial and prefecture governments are not incentivized to prioritize equalization transfers to the lower government levels, county and township governments particularly in less developed localities lack the necessary resources to comply with all expenditure responsibilities assigned under national policies. In the absence of adequate performance monitoring system at the sub-national levels as well as appropriate equalization fiscal transfers across localities within as well as across provinces, the fiscal constraints at the local levels generate an uneven progress in health system reform implementation."[29]

"The financial sustainability of the system is threatened by problems related to the country's economic crisis, fiscal evasion, corruption in the management of the Subsidized Regimen and inaccurate economic forecasts on which the expansion of coverage has been based. The increase in unemployment and the temporary and informal nature of employment have contributed to the depletion of the system's capacity to accrue resources, making it impossible to achieve universal coverage."[30]

"This failure to progress towards universalism can be explained by the unrelenting pressure on the health system as a result of budget cuts since 2009 and by the lack of clarity on the exact form of universalism espoused and the mechanisms to achieve it."[31]

"Unaffordability of the increased premium rates limited access of certain citizen groups to the entitlements."[33]

"Study participants considered the new premium rates for the informal sector unfair and unaffordable as they were fixed over quite large income ranges. Premium contributions for the formal sector were, however, graduated depending on the salary range. Our review of the new premiums rates showed that they were regressive as low-income earning population groups in

both the formal and informal sectors contributed more of their income towards the premiums than higher-income earning groups."[33]

"Unaffordability of the new premiums was considered a barrier to enrolment and hence a barrier to access to needed care particularly for the unemployed; those living in rural and marginalised areas; the youth of 18 years and above but not enrolled in school; the elderly; people living with disabilities and those in the informal sector with meagre and unstable earnings."[33]

"Unequal distribution of entitlements in the new benefit packages across different population groups limited citizen's access to the entitlements."[33]

"The new provider payments did not incentivize equity, efficiency and quality healthcare service provision due to perceived inadequacy in payment rates. Both public and private providers indicated that the capitation rates offered for outpatient services were inadequate as they did not take into consideration the actual costs of services or the number of times an NHIF beneficiary would visit a health facility."[33]

"NHIF beneficiaries seeking care in private facilities felt that the delayed reimbursements by NHIF had incentivized providers to: 1) introduce co-payments, 2) deny or ration services offered to them, 3) treat them with less respect, or 4) expose them to longer waiting times than patients with other forms of insurance or cash paying patients."[33]

"The higher premium rates were unaffordable and regressive for the poor, elderly, people living with disabilities, unemployed and informal sector workers with meagre and unstable earnings. This policy design created a financial barrier to enrolment and led to attrition."[33]

"Differences in the benefit package between the national scheme and enhanced schemes, a policy design issue, led to inequities in access to services and OOP payments for the services not covered. Differences in benefit package design led to differences in financial protection where generous benefit packages are associated with lower OOP payments."[33]

Health system structure and infrastructure:

"Latin American health systems have intrinsic weaknesses, with fragmentation of organisation and service delivery, segmentation of financing, and a poorly regulated private sector, presenting challenges to the development of equitable and efficient health systems."[22]

"The studies highlight the need to pay attention to both supply side and demand side factors in UHC reforms: countries like Ghana, Kenya, and India which are struggling with inadequate service delivery capacities and quality of care issues, are also having difficulty making their

UHC schemes attractive to the population, even with an ostensibly extensive and generous benefits package as in Ghana's case."[23]

"Indeed, the low UHC coverage indices of most of these countries are often a reflection of 'generous' benefits packages on paper but which are in practice not fully available to the beneficiaries, especially those in rural and peri-urban areas. A lesson here is that substantial supply-side investments are essential to making progress on delivering the full promise of UHC to its intended beneficiaries."[23]

"The dissatisfaction of Italians with respect to the efficiency and quality of their health care ranks the highest in Europe. Largely as a consequence of this dissatisfaction, recourse to the private market for services has increased steadily in the last few years."[24]

"Unavailable or overcrowded services either left patients unattended or they turned to other providers. If insured at the SSC, people may have gotten treatment there. If not, private providers were the only option."[26]

"Queues at the public hospitals led patients to choose private clinics. … Better off inhabitants preferred private providers, avoiding waiting time and extensive documentation."[26]

"Thus, out-of-pocket expenditure existed, despite the abolition of fees, when patients were forced to use private services or purchase drugs when they were unavailable in the public system. … Abolition of user fee policies should thus not be confused with a no-cost health care system."[26]

"Southern NHSs were already mature social insurance health care systems when the transformation took place. This entails greater challenges and difficulties than when such transformation is imposed on a very primitive system."[28]

"Perceived inadequacy of, and delayed NHIF reimbursements, a policy implementation issue, led to preferential treatment of privately insured and/or uninsured cash-paying patients over NHIF beneficiaries particularly in private hospitals. The provider incentives embodied in payments systems influence provider behaviour in treatment decisions which in turn affect equity in access to needed services, quality and efficiency of service provision."[33]

"Reforms of benefit packages should also inform infrastructure developments, failure to which makes the benefit package merely a wish list, with limited access to actual services and limited financial risk protection."[33]

"NHIF beneficiaries seeking care in private facilities felt that the delayed reimbursements by NHIF had incentivized providers to: 1) introduce co-payments, 2) deny or ration services

offered to them, 3) treat them with less respect, or 4) expose them to longer waiting times than patients with other forms of insurance or cash paying patients."[33]

"While the new reforms included new benefit entitlements, they were not accompanied by reforms on infrastructure improvement."[33]

"Quality of care particularly in public hospitals was compromised by lack of accompanying reforms on quality and infrastructure improvement- a weakness in the design and implementation of the new policies."[33]

"The participants frequently emphasized on the lack of effective cooperation between the public and private sectors as the main obstacle to the successful implementation of the reforms. They believed that improper implementation of primary health care, conflict of interest, and inadequacies in the registration system hindered the referral system and in turn, contributed to the fragmentation of the healthcare system."[34]

"An important determinant affecting HSR in Iran has been a chaotic healthcare system. … Fragmentation of the Iranian healthcare system was another concept identified by participants as a major challenge for any reform."[34]

"Although they are made at the central level, national policies often have to be implemented by subnational entities that are largely autonomous. This often leads to multiple sources of fragmentation and much potential for the duplication of efforts and consequent inefficiencies."[35]

"In the Russian Federation, formal and informal out-of-pocket payments still create barriers to accessing care for certain groups of the population – even though the entire population is now covered by mandatory health insurance and the state medical benefit package."[35]

"Despite the government's policy encouraging a tiered delivery system anchored by PHC, actual implementation deviates substantially from the ideal model; without a functioning PHC system, medical alliances in China are predominantly led by hospitals."[40]

Re health systems that strengthened Primary Healthcare, often as part of, or a precursor to, UHC reforms:

"Most of the study countries introduced comprehensive primary health care to improve access to health services for many millions of people, with improved health outcomes and financial risk protection."[22]

"Starting with the 1990s, a comprehensive primary health-care model underpinned by biopsychosocial approaches began to emerge in the Latin American countries studied. What followed was the development of comprehensive primary health care, public health

interventions that incorporated and intersectoral and asserted a rights-based collaboration, as approach to health, citizen participation, community empowerment, positioned primary health care the platform for achieving equity and universal health coverage."[22]

"Countries must build robust healthcare systems founded on primary healthcare (PHC) to ensure access to quality preventative and curative healthcare. Over 134 nations committed to making PHC the cornerstone of their health system through the 2018 Declaration on PHC in Astana."[38]

"If the responsibility for the teams lay with an organization that primarily emphasized curative care, preventive care would likely not play an equal role. This horizontal integration of service delivery at all structural levels of the health care system helps drive the success of primary care in Costa Rica."[39]

"Such a population health approach underlies the horizontal integration of preventive, curative, and public health services within each EBAIS [integrated care] team. By knowing exactly who they must care for, how well they are providing care to that population, and what their plan is for improving that care in the coming year, members of a team can effectively direct their resources and efforts to maximizing population health."[39]

Political Commitment:

"The sociopolitical context of each country affects the trajectory of implementation and change significantly."[12]

"Legislation can preserve key reform components through future political fluctuations."[12]

"Our contention is that achieving the principle of universality in healthcare systems is less of a technical matter and more a political project."[25]

"Healthcare systems that aim to achieve universality or bring about sustainable pro-equity change cannot do so unless the broader socio-political context is conducive to such a change."[25]

"The political context determines the nature of the policies, while other essential elements are the constitutional and legal changes that explicitly mention universality, broad public participation in health systems, and health as a social right to be provided by the state as principles guiding the development of reformed health systems."[25]

"The strong politicisation of the healthcare system has made the decision-making process very difficult. According to the participants, in such a politicized environment, no one can make a long-term decision or plan far ahead."[34]

"Implementation of health financing reforms for Universal Health Coverage (UHC) is inherently political."[42]

Communication and Relationships:

"We conclude that an understanding of the state-society relations is vitally important to assess the conduciveness of any efforts to reform healthcare systems."[25]

"Furthermore, insufficient information and communication with stakeholders and the population concerning the reform process was perceived in rural Ecuador as well as in several African countries."[26]

"The government's responsibility to properly and continuously inform all citizens about their reforms cannot be overemphasized. Some of the confusion expressed could probably have been avoided by better coordination and communication from those responsible for implementation of the reform."[26]

"Some [research participants] felt that knowledge and awareness of the new reforms by certain population groups was limited by: 1) the use of complex language (such as medical terms) to describe the services offered, 2) vagueness of the benefit package where services were presented in broad categories making it difficult for users to know what specific services they are entitled to, 3) limited geographical coverage of communication and sensitization campaigns and, 4) limited knowledge and awareness among some healthcare providers who were gatekeepers for access to these services."[33]

"China's effort to engage the domestic academic and research community, and international agencies has generated a strong evidence and technical basis on which the government has built China's health system reform."[29]

"China's health system has not engaged sufficiently with service providers, especially the public hospitals, during the process. That may be reflected in the lack of due process in reforming public hospitals over the past 3 years, as the buying in of the reform by the Chinese hospital sector has not be clearly evident."[29]

"The lack of clarity on the mechanism and route to achieve UHI also meant that it failed to gain public or political support."[31]

"Setting a clear, compelling vision enabled leaders to mobilise stakeholder commitment."[38]

"While other Latin American countries implemented health reforms solely in a top- down direction, Costa Rica's strategy of deep community engagement strengthened their reform by

creating transparency and building buy-in. To overcome initial resistance, leaders applied methods described in change management literature."[38]

"Health leaders convened to define a vision for the new PHC model: to provide equitable, comprehensive healthcare, equally emphasising promotive, preventative and curative health services. This compelling vision was critical for building buy- in from diverse stakeholders. Based on the vision and learnings, health leaders developed a proposal for three PHC reforms."[38]

"Historical animosity as well as different pay scales and benefit packages led to initial widespread resistance among staff ... [but] negotiations enabled staff to resolve areas of disagreement and buy into the reform."[38]

"Ideas are powerful, because they embody the narratives, the metaphors that shape how UHC is perceived, and how it is discussed and popularly represented. The power of the ideas rests on the notion that symbols or a persuasive story can be more important than material or objective fact."[42]
